# Assessing usability and satisfaction of telephone-based psychiatry consultations in Oman: a cross-sectional study

**DOI:** 10.3389/fdgth.2025.1563180

**Published:** 2025-06-04

**Authors:** Tamadhir Al-Mahrouqi, Fatema Al-Sabahi, Mohammed Al-Alawi, Munira Al Rubkhi, Marwa Al Abdali, Muna Al Salmi, Tharaya Al-Hashemi, Rahma Al Nuumani, Fatma Al Balushi, Rashid Al Zaidi, Samir Al-Adawi, Sachin Jose, Hassan Mirza

**Affiliations:** ^1^ Department of Behavioral Medicine, Sultan Qaboos University Hospital, College of Medicine and Health Sciences, Sultan Qaboos University, Muscat, Oman; ^2^ College of Medicine and Health Sciences, Sultan Qaboos University, Muscat, Oman; ^3^ Department of Psychiatry, Al Masarra Hospital, Ministry of Health, Muscat, Oman; ^4^ Research Department, Oman Medical Specialty Board, Muscat, Oman

**Keywords:** patient satisfaction, usability, health care accessibility, telehealth, telepsychiatry, psychiatric consultation

## Abstract

**Background:**

Telehealth has become a valuable tool for providing health services remotely through digital communication technologies. Therefore, assessing patients' satisfaction and the utility of this tool is crucial for future implementation and development.

**Methods:**

A cross-sectional survey study was conducted at Al Masarra Hospital between January 1st, 2023, and June 30th, 2023, with adults recruited from the general adult psychiatry clinic and received at least 3 previous telephone-based psychiatry consultations. Data were then analyzed using the Chi-square test and a multivariate binary logistic regression.

**Results:**

Among 222 patients (a mean age = 37.72 years), the majority (77.5%) were found to have an overall satisfaction with the service. Most participants agreed that telehealth improves access to healthcare services (65.2%), saves time traveling (78.4%), and meets their healthcare needs (75.7%). A total of 22.5% of participants reported dissatisfaction with the service. Significant predictors of satisfaction included sex, employment status, and monthly income. Females were more likely to be satisfied with telephone-based psychiatric consultations (Adjusted OR = 2.525, *p* = 0.030). Homemakers or unemployed participants were more likely to report satisfaction (Adjusted OR = 7.838, *p* = 0.009), as were those earning between 150 and 499 OMR (Adjusted OR = 10.967, *p* = 0.001) and ≥500 OMR (Adjusted OR = 46.312, *p* < 0.001).

**Conclusion:**

Telephone-based psychiatry consultations demonstrated high usability, satisfaction, and accessibility among study participants. Sex, employment status, and monthly income significantly influenced satisfaction levels. These findings highlight the potential of telehealth to bridge gaps in mental healthcare delivery, particularly for underserved populations in Oman. It is important to consider, however, the issues that might arise during these consultations that lead to dissatisfaction in some patients, and find suitable solutions for both the patients and the healthcare providers.

## Introduction

Telepsychiatry is a subset of telehealth that offers psychiatric care services using various communication technologies, including telephone or video calls, text messaging, emails, and fax (1, 2). While the utility of telepsychiatry was established long before the COVID-19 pandemic, its demand has increased significantly during and after the pandemic. This increase in demand is attributed to the adverse effects of the pandemic and lifestyle changes, which have elevated rates of depression, anxiety, and other psychological repercussions worldwide (3, 4). Therefore, the importance of telepsychiatry in providing psychiatric services has increased while maintaining social distancing and controlling the spread of infection (3–5). The introduction of telepsychiatry in Oman began in non-governmental clinics, which adopted various telepsychiatry modalities such as telephone calls, video conferencing, and messaging services. However, during the pandemic, government hospitals such as Al-Masarah Hospital also embraced telepsychiatry, with a particular emphasis on telephone consultations to provide mental health care to patients.

Telepsychiatry has become a valuable tool for providing health services remotely through digital communication technologies (6). Its utility can serve various aspects of the health field, including disease detection, disaster management, healthcare delivery, and educational and administrative purposes (6). In addition, it started initially with telephone consultations; however, it quickly developed, especially during the COVID-19 pandemic, to include various forms such as video conferencing, text messages, and email newsletters (1, 6). This helped provide flexibility and convenience to patients and healthcare providers (2). Additionally, several studies have shown that telehealth during the COVID-19 pandemic has helped control disease spread while providing the necessary medical care to healthcare users (1, 6–8).

Although telepsychiatry has received more attention in the literature and research after the pandemic, more studies are needed to explore its efficacy. Regarding the patient's experience, the literature has shown general satisfaction among patients and their caregivers with telepsychiatry in providing the needed care (9). A systematic review has shown that 23 out of 31 quantitative studies reported that the range of patient experience with telepsychiatry was good to excellent. Furthermore, a randomized controlled study comparing patient satisfaction with telepsychiatry and face-to-face consultations revealed the same satisfaction among the two groups. However, patients using telepsychiatry consultations found that it was less supported and encouraged compared to patients attending face-to-face consultations. Furthermore, a cross-sectional study carried out at a university hospital in Riyadh found that patients reported high satisfaction during telephone psychiatry consultations due to the convenience, privacy, and skill of the clinicians (10). And nearly 50% of the participants reported interest in further use of telepsychiatry in the future (10).

Furthermore, in Oman, there have been limited studies exploring the use of telehealth in general and various forms of telepsychiatry in particular. For example, a recent qualitative study conducted in Oman reported positive reactions and satisfaction among psychotherapists when using video conferencing to deliver Cognitive Behavioral Therapy (CBT) and Acceptance and Commitment Therapy (ACT) (5). In addition, a randomized controlled trial conducted in Oman aimed to compare different telepsychiatry modalities for patients experiencing depression and anxiety during the COVID-19 pandemic. The results indicated that video-conferencing with an online psychotherapist guide outperformed self-help emails (4). In Oman, there are two studies investigated the effectiveness of telephone consultations as a prominent form of telepsychiatry in the government sector (5, 11). These qualitative studies delved into psychiatrists’ experiences with conducting telephone-based psychiatric consultations (5, 11). The results indicated that psychiatrists generally expressed satisfaction with the convenience and flexibility of telephone consultations (5, 11). However, they also raised concerns about issues related to confidentiality and the absence of visual cues (5, 11). Additionally, the psychiatrists who participated in one of these studies offered recommendations for the implementation of an electronic health system that promotes communication between various levels of healthcare and the incorporation of electronic prescription capabilities that can improve the effectiveness of telepsychiatry services across the country (11). It is also essential to explore the patients’ point of view on the usefulness of telephone consultations. The study also provides some information on the usefulness of telephone psychiatric consultations for patients, but from the point of view of participating psychiatrists.

Recent studies across the Gulf region have explored patient satisfaction and the implementation of telepsychiatry services. In the United Arab Emirates (UAE), a cross-sectional study reported that 83.1% of telemedicine users expressed overall satisfaction with their experience, indicating a positive reception of telehealth services (12). Similarly, the Dubai Health Authority observed that over 78% of scheduled telehealth visits were completed, suggesting effective utilization and patient engagement (13). Conversely, a study in Saudi Arabia identified barriers to telemedicine adoption, including technological illiteracy and potential discomfort from camera use, which limited widespread acceptance (14). These contrasting findings highlight the variability in telehealth adoption and satisfaction within the Gulf region. Given the unique cultural and infrastructural context of Oman, there is a pressing need for localized research to understand the specific factors influencing telepsychiatry effectiveness and patient satisfaction in the country.​ Therefore, in this cross-sectional study, our primary objective is to examine the patients’ experiences, opinions, and levels of satisfaction with the telephone psychiatric consultations they have received. By doing so, we aim to gather a better understanding of the effectiveness and drawbacks of these consultations from the patient's point of view, which can complement the insights gained from the healthcare providers’ perspective.

### Objectives

1. Assess satisfaction with the use of telephone psychiatric consultations among study participants.
2. Explore the factors that are associated with satisfaction with the use of telephone-based psychiatrist consultations among the study participants.

## Materials and methods

### Study design, setting & participants

This was a cross-sectional study conducted at Al Masarra Hospital in Muscat, Sultanate of Oman. The Al Masarra Hospital is the only governmental tertiary hospital for psychiatry in the Sultanate of Oman, with a bed capacity of 245 beds. Psychiatry services are classified into four sections. General Adult Psychiatry, Geriatric Psychiatry, Child and Adolescent Psychiatry, and Forensic Psychiatry. Telephone psychiatry consultations are provided by the General Adult Psychiatry Section and the Child & Adolescent Psychiatry Section.

This study included a cohort of psychiatric patients who received follow-up care in the general adult psychiatry clinic and received a telephone-based psychiatry consultation from January 1, 2023, to June 30, 2023.

### Inclusion and exclusion criteria of study participants

All adult patients (aged 18–60 years) who followed up in the general adult psychiatry clinic and received at least 3 previous telephone-based psychiatry consultations during the duration of the study period were invited by research team members to participate in the study when they attended a scheduled in-person consultation. Patients with acute psychosis, intellectual disability, or medical or psychiatric illness contributing to cognitive impairment were not invited to participate. Patients who refused to sign the informed consent or provided incomplete forms were excluded. The study population meeting this inclusion criteria during the study period was 430 individuals, and they were all invited to participate.

### Data collection

A paper-based and electronic version of the data collection tool was developed using Google Forms. During the data collection period from the 1st of September to the 31st of October, research team members approached patients who met the inclusion criteria while they were attending in-person visits at the clinic. The study was explained to each eligible participant individually during their waiting time in the clinic. Patients were then invited to complete either the paper format or the electronic version of the questionnaire. For those who preferred the electronic format, a barcode linking directly to the online questionnaire was provided to facilitate easy access and participation.

### Study instruments

#### Sociodemographic questionnaire

The questionnaire collected information about the participant's age, sex, nationality, marital status, parenting status, hospital distance (Km), highest educational attainment, employment status, monthly financial income, diagnosis, and years since the patient was diagnosed.

#### Telehealth usability questionnaire (TUQ)

The Telehealth Usability Questionnaire (TUQ) comprises 21 items designed to assess the efficacy of treatment provided through remote rehabilitation services, software or systems. Developed by Parmanto et al. in 2016 (15), the TUQ evaluates usability across multiple dimensions, including usefulness (3 items), ease of use and learnability (3 items), interface quality(4 items), interaction quality(4 items), reliability(3 items), satisfaction, and future use (4 items). The questionnaire comprehensively addresses key aspects of usability. Respondents rate their agreement on a 7-point Likert scale (1 = disagree, 7 = agree). The total score is obtained by summing the responses to the 21 items. Previous studies have reported the validity of the content of the questionnaire items (15, 16), and the TUQ demonstrates excellent reliability, specifically in terms of internal consistency, as indicated in previous research (15, 16). The face validity of the Arabic language was previously established (14).

### Sample size

The sample size for this study was determined using the OpenEpi epidemiological tool. The target population included 430 individuals between the ages of 18 and 60 years who met the study's inclusion criteria. Eligibility was defined as having completed at least three prior telepsychiatry consultations via phone calls and attending an in-person consultation during the study period.

Based on previous literature, an anticipated satisfaction rate of 70% for telepsychiatry was used, with a 5% margin of error and a 95% confidence level (14, 17). These parameters yielded a minimum required sample size of 185 participants. However, to maximize participation and data reliability, all 430 eligible individuals were invited to participate. A total of 222 participants responded to the survey, exceeding the minimum sample size requirement.

### Statistical analysis

Continuous variables were presented as mean, median, standard deviation, and interquartile range, whereas categorical variables were presented as frequency and percentage. Mean scores between two groups were compared using the independent samples *t*-test. The association between two categorical variables was assessed using either Fisher's exact test or the Likelihood ratio test, as appropriate. A multivariate binary logistic regression analysis was performed to determine the independent predictors of the overall satisfaction for the telephone-based telepsychiatry services among participants. Odds ratios were reported with a 95% confidence interval. A *P*-value less than 0.05 was considered statistically significant. All the analysis was carried out in IBM SPSS Statistics (IBM Corp. Released 2022. IBM SPSS Statistics for Windows, Version 29.0. Armonk, NY: IBM Corp).

### Ethical approval

The Medical Research Ethics Committee approved the study in the Ministry of Health (MoH/CSR/23/27816). All participants contributed to the study based on their willingness to participate. The right to withdraw from the research study was explained to all participants at any time.

## Results

As shown in Table 1. The study included 222 participants, with a mean age of 37.72 years (SD ± 9.97). Slightly more than half of the participants were male (53.6%), while females constituted 46.4%. All the participants were Omani nationals. Regarding marital status, nearly half of the participants were married (48.6%), followed by those who were single (39.2%), divorced or widowed (12.2%). A significant portion of participants (62.6%) reported living more than 70 km away from Al Masarra Hospital, with the remaining 37.4% residing within 70 km.

**Table 1:** Socio-demographic characteristics of the participants.

Variable	*n* (%)
Age, mean ± SD	37.72 ± 9.97
Sex	
Male	119 (53.6)
Female	103 (46.4)
119 (53.6)	
103 (46.4)	87 (39.2)
Married	108 (48.6)
Divorced/Widowed	27 (12.2)
Parental status	
Have children	122 (55.0)
Do not have children	100 (45.0)
Distance between Hospital and your home	
Less than 70 km	83 (37.4)
More than 70 km	139 (62.6)
Education	
Less than high school diploma	93 (41.9)
High school diploma certificate	93 (41.9)
Bachelors & above	36 (16.2)
Employment status	
Student	16 (7.2)
Job seeker	36 (16.2)
Home maker or unemployed	75 (33.8)
Self-employed or business owners	9 (4.1)
Employed	86 (38.7)
Monthly income	
No income	64 (28.8)
<150 OMR	26 (11.7)
150–499 OMR	71 (32.0)
≥500 OMR	61 (27.5)

Educational attainment was fairly evenly distributed between those with less than a high school diploma (41.9%) and those with a high school diploma (41.9%), while a smaller proportion had a bachelor's degree or a master's/PhD degree (16.2%). Employment status revealed that 38.7% were employed, followed by homemakers or unemployed individuals (33.8%), job seekers (16.2%). In terms of financial income, 28.8% reported having no income, while 32.0% earned between 150 and 499 OMR per month. Smaller proportions had an income of 500 OMR or more (27.5%), and a minority (11.7%) earned less than 150 OMR monthly.

Table 2 shows the internal consistency of the Telehealth Usability Questionnaire (TUQ) was excellent, with an overall Cronbach's alpha of 0.957. Among the subscales, satisfaction demonstrated the highest reliability (*α* = 0.930), followed by ease of use (*α* = 0.874), usefulness (*α* = 0.807), and effectiveness (*α* = 0.804). The reliability subscale showed acceptable internal consistency (*α* = 0.694).

**Table 2:** Internal consistency of the TUQ subscales.

Variable	Cronbach's alpha
Overall	0.957
Subscale	
Usefulness	0.807
Ease of use	0.874
Effectiveness	0.804
Reliability	0.694
Satisfaction	0.930

Table 3 shows the TUQ scores by subscales. The mean overall TUQ score was 108.59 ± 26.53, with a median score of 117 (range: 21–147). Among the subscales, the ease of use subscale had the highest mean score (30.63 ± 7.92), with a median of 32 (range: 6–42). The effectiveness subscale followed with a mean score of 25.29 ± 6.56 and a median of 27 (range: 5–35). The satisfaction subscale recorded a mean of 21.60 ± 6.23, with a median of 24 (range: 4–28). The usefulness and reliability subscales had comparable mean scores of 15.78 ± 4.43 and 15.30 ± 4.12, respectively, both with medians of 17 (ranges: 3–21 for usefulness and 3–21 for reliability).

**Table 3:** TUQ scores by subscales.

Variable	TUQ score	
	Mean ± SD	Median (Min.–Max.)
Overall	108.59 ± 26.53	117 (21–147)
Subscale		
Usefulness	15.78 ± 4.43	17 (3–21)
Ease of use	30.63 ± 7.92	32 (6–42)
Effectiveness	25.29 ± 6.56	27 (5–35)
Reliability	15.30 ± 4.12	17 (3–21)
Satisfaction	21.60 ± 6.23	24 (4–28)

Table 4 presents the distribution of respondents’ agreement levels across each telehealth questionnaire item, reflecting perceptions of usability, effectiveness, and satisfaction with the system. 78.4% agreed that telehealth saved them travel time, and 65.2% that it improved their access to care. Three-quarters found the system easy to learn (75.2%) and reported they would use it again (73.4%) and that it was an acceptable way to receive services (74.8%). Clear audio quality scored highest (79.3% agreement), while visual equivalence to in-person visits (56.8%) and the system's ability to provide every desired function (59.9%) received comparatively lower but still majority agreement. Overall satisfaction peaked at 77.5%, underscoring broad user endorsement of this telehealth platform. The 95% confidence interval for overall satisfaction ranged from 71.41% to 82.97%, indicating a high level of positive reception toward telephone-based psychiatry consultations.

**Table 4:** Respondents’ level of agreement with each questionnaire item.

Item	Disagree^a^	Neither agree nor disagree	Agree^b^
	*n* (%)	*n* (%)	*n* (%)
Telehealth improves my access to healthcare services	52 (23.5)	25 (11.3)	144 (65.2)
Telehealth saves me time traveling to a hospital or specialist clinic	29 (13.1)	19 (8.6)	174 (78.4)
Telehealth provides for my healthcare need	29 (13.1)	25 (11.3)	168 (75.7)
It was simple to use this system	23 (10.4)	64 (28.8)	135 (60.8)
It was easy to learn to use the system	19 (8.6)	36 (16.2)	167 (75.2)
I believe I could become productive quickly using this system	44 (19.8)	40 (18.0)	138 (62.2)
The way I interact with this system is pleasant	40 (18.0)	34 (15.3)	148 (66.7)
I like using the system	34 (15.3)	43 (19.4)	145 (65.3)
The system is simple and easy to understand	45 (20.3)	27 (12.2)	150 (67.6)
This system is able to do everything I would want it to be able to do	53 (23.9)	36 (16.2)	133 (59.9)
I can easily talk to the clinician using the telehealth system	36 (16.2)	37 (16.7)	149 (67.1)
I can hear the clinician clearly using the telehealth system	27 (12.2)	19 (8.6)	176 (79.3)
I felt I was able to express myself effectively	27 (12.2)	45 (20.3)	150 (67.6)
Using the telehealth system, I can see the clinician as well as if we met in person	56 (25.2)	40 (18.0)	126 (56.8)
I think the visits provided over the telehealth system are the same as in-person visits	49 (22.1)	37 (16.7)	136 (61.3)
Whenever I made a mistake using the system, I could recover easily and quickly	36 (16.2)	52 (23.4)	134 (60.4)
The system gave error messages that clearly told me how to fix problems	38 (17.1)	36 (16.2)	148 (66.7)
I feel comfortable communicating with the clinician using the telehealth system	42 (18.9)	21 (9.5)	159 (71.6)
Telehealth is an acceptable way to receive healthcare services	36 (16.2)	20 (9.0)	166 (74.8)
I would use telehealth services again	34 (15.3)	25 (11.3)	163 (73.4)
Overall, I am satisfied with this telehealth system	28 (12.6)	22 (9.9)	172 (77.5)

^a^ Disagree—strongly disagree + Disagree + somewhat disagree.

^b^ Agree—strongly agree + agree + somewhat agree.

Table 5 shows the univariate and multivariate analyses that identified the predictors of overall satisfaction with telephone-based psychiatry consultations. Sex, Employment status, and monthly income were emerged as significant factors. Female patients are significantly more satisfied with telephone-based psychiatric consultations than male patients, with satisfaction levels being approximately 2.5 times higher (OR = 2.525, 95% CI: 1.094–5.832, *P* = 0.030). Individuals actively seeking employment report five times higher satisfaction compared to those who are currently employed (OR = 5.091, 95% CI: 1.023–25.347, *P* = 0.047). Similarly, unemployed individuals or homemakers experience eight times greater satisfaction than employed individuals (OR = 7.838, 95% CI: 1.655–37.117, *P* = 0.009). Patients earning a monthly income between 150 and 499 OMR report 11 times more satisfaction than those with no income (OR = 10.967, 95% CI: 2.745–43.824, *P* = 0.001). Notably, patients with a monthly income of 500 OMR or more show the highest satisfaction, being 46 times more likely to be satisfied compared to those with no income (OR = 46.312, 95% CI: 7.851–273.205, *P* < 0.001). Other variables, such as age, marital status, distance from the hospital, and educational attainment, did not show significant associations with overall satisfaction.

**Table 5:** Univariate and multivariate analysis to determine the independent predictors of overall satisfaction with telephone-based psychiatry consultations.

Variable	Overall satisfaction		*P*-value	β	Adjusted odds ratio (Adj. OR)	95% CI for Adj. OR		*P*-value
	Not satisfied (*n* = 50)	Satisfied (*n* = 172)						
	*n* (%)	*n* (%)				Lower	Upper	
Age, mea*n* ± SD	37.48 ± 11.07	37.78 ± 9.66	0.850	−0.025	0.976	0.932	1.021	0.288
Sex								
Male (Reference)	29 (24.4)	90 (75.6)	0.522		1.000			
Female	21 (20.4)	82 (79.6)		0.926	2.525	1.094	5.832	0.030^a^
Marital status								
Single (Reference)	21 (24.1)	66 (75.9)	0.743		1.000			
Married	22 (20.4)	86 (79.6)		−0.476	0.621	0.156	2.470	0.499
Divorced/Widowed	7 (25.9)	20 (74.1)		−0.825	0.438	0.089	2.145	0.309
Parental status								
Have children (Reference)	24 (19.7)	98 (80.3)	0.333		1.000			
Do not have children	26 (26.0)	74 (74.0)		−0.789	0.454	0.123	1.675	0.236
Distance between hospital and your home								
Less than 70 km (Reference)	23 (27.7)	60 (72.3)	0.184		1.000			
More than 70 km	27 (19.4)	112 (80.6)		0.699	2.012	0.954	4.244	0.066
Education								
Less than high school diploma (Reference)	22 (23.7)	71 (76.3)	0.361		1.000			
High school diploma certificate	23 (24.7)	70 (75.3)		−0.180	0.836	0.360	1.938	0.676
Bachelors & above	5 (13.9)	31 (86.1)		0.009	1.009	0.279	3.653	0.989
Employment status								
Employed (Reference)	17 (19.8)	69 (80.2)			1.000			
Student	5 (31.3)	11 (68.8)	0.779	1.200	3.320	0.517	21.313	0.206
Job seeker	9 (25.0)	27 (75.0)		1.627	5.091	1.023	25.347	0.047^a^
Home maker or unemployed	16 (21.3)	59 (78.7)		2.059	7.838	1.655	37.117	0.009^a^
Self-employed or business owners	3 (33.3)	6 (66.7)		−0.086	0.918	0.145	5.803	0.928
Monthly income								
No income (Reference)	21 (32.8)	43 (67.2)	0.006^a^		1.000			
<150 OMR	9 (34.6)	17 (65.4)		0.238	1.268	0.414	3.883	0.677
150–499 OMR	14 (19.7)	57 (80.3)		2.395	10.967	2.745	43.824	0.001^a^
≥500 OMR	6 (9.8)	55 (90.2)		3.835	46.312	7.851	273.205	<0.001^a^

^a^ Statistically significant, Test: Independent samples t-test, Fisher's exact, Likelihood ratio, Multivariate binary logistic regression analysis.

## Discussion

This cohort study has explored the satisfaction of adult patients attending Al Masara Hospital with telehealth services provided. The overall results show a positive perception of this service. In this section, we will discuss our results in contrast with previous studies.

Overall satisfaction among participants was high with the telehealth service provided in Al Masara Hospital, which is in line with other studies that have reported similar results (10, 18). Most patients found telehealth consultation a beneficial, convenient, and cost-effective tool (11, 19). A study conducted in Pennsylvania examining the reliability of administering the adult autism assessment via video consultation found high levels of participant satisfaction (2). On the other hand, psychiatrists view it as a valuable tool for follow-up and increasing patients’ adherence to attending consultations (11).

Regarding accessibility and usability, the majority of the study participants agreed that telehealth has improved their access to mental health care services. Moreover, over three-quarters agreed that telehealth is a time-efficient element that meets their needs. These results were consistent with one of our previous qualitative studies that reported that telehealth eases patients’ accessibility regarding appointment scheduling and fast follow-up (11). Additionally, it has been shown that telehealth eliminates the time and cost of transportation for patients compared to traditional consultations (11). Therapists have reported similar benefits, such as telehealth making reaching out to patients in rural areas easier (18). A qualitative explorative study was conducted at Al Masarra Hospital in 2023 to examine psychiatrists’ experience with telehealth, and it was reported that most psychiatrists found telehealth convenient and time-efficient (11). On the other hand, the same study raised concerns regarding technical support and tools provided, as psychiatrists reported that the significant challenge they may face is the limited availability of external phone lines, which could negatively impact the accessibility to the conduct of the service (11).

Moreover, learnability is one of the essential elements that this study has examined. It has been shown that most participants think that telehealth is easy to understand and that the system is easy to learn. However, a cross-sectional study in Riyadh showed that patients strongly disagreed with using technical terms during telehealth consultations (10). Moreover, psychiatrists found it challenging to communicate over the phone due to the absence of visual cues and the inability to analyze patients’ facial expressions and body language (11). Therefore, it is vital to consider the communication gaps that could occur during telehealth consultations.

Regarding the quality of interaction, most of the participants in this study were satisfied with the quality of interaction with their psychiatrists during telehealth consultations. The majority reported that they could express themselves effectively and that they could hear their clinicians effectively. In addition, the larger proportion of patients agreed that they could see the clinicians as if they met in person, unlike a previous cross-sectional study that showed that patients were less satisfied with the quality of interaction during telephone-based consultations. The authors justified this result as patients could not receive non-verbal feedback during the consultation due to the absence of the visual element throughout the consultation (18). This highlighted the significance of rapport and nonverbal cues during psychiatric consultations (18). Similarly, the lack of visual cues was a concern reported by psychiatrists in our previous study (11). Psychiatrists found it challenging to communicate over the phone due to the absence of visual cues and the inability to analyze patients’ facial expressions and body language.

This study did not find a relationship between demographic characteristics and the level of satisfaction. A previous study also failed to report a link between patients’ demographics and satisfaction scores (10). Additionally, another study found a relationship between the level of patients’ satisfaction and how far they live from the hospital. Patients with greater distance reported higher satisfaction with telehealth consultation as they considered it more convenient (18). No other significant effect of demographic characteristics on patients’ satisfaction levels was found in this study (18).

While this study highlights the benefits of telehealth, addressing technical challenges is crucial to fully realize its potential. Issues such as inconsistent connectivity, lack of integration with electronic health records, and the absence of visual cues during consultations can hinder the effectiveness of telehealth services (20). Future efforts should focus on improving technological infrastructure, developing user-friendly platforms, and providing training for both patients and providers (20). By addressing these challenges, telehealth can further expand access to mental health services, reduce stigma, and minimize logistical barriers such as commute and waiting times, paving the way for a more inclusive and efficient psychiatric care system (20).

### Limitations

This study has several limitations that should be acknowledged. First, the cross-sectional design limits the ability to establish causal relationships between variables. Second, while the study utilized the validated Arabic version of the Telehealth Usability Questionnaire (TUQ), the reliance on self-reported data may have introduced response bias. Thirdly, the study excluded individuals with acute psychosis or cognitive impairments, which limits the generalizability of the findings to all psychiatric patient populations. Finally, the classification of monthly income used in this study was based on locally relevant but non-standardized thresholds determined by the research team. This may limit comparability with other national or international studies. Future research would benefit from using standardized income brackets based on official Omani statistics or globally recognized socioeconomic indices to improve consistency and generalizability.

### Strengths of the study

This study is strengthened by its setting in Al Masarra Hospital, the only tertiary psychiatric center in Oman, ensuring a well-defined and relevant population. The use of a validated and reliable instrument, the Arabic version of the Telehealth Usability Questionnaire (TUQ), adds methodological rigor and enhances the accuracy of usability assessment. Additionally, the study employed clearly defined inclusion and exclusion criteria, contributing to the internal validity. The use of both paper and digital data collection methods improved participant accessibility and response rates, thereby increasing the representativeness of the findings.

## Conclusions

This study highlights the high usability and satisfaction levels among psychiatric patients utilizing telephone-based consultations at Al Masarra Hospital. Telehealth has proven to be a convenient and accessible tool, particularly for individuals residing in remote areas, by reducing commute times and associated costs. However, addressing technical challenges such as connectivity issues and the absence of visual cues is essential for enhancing the overall effectiveness of telepsychiatry. By addressing these limitations and expanding telehealth services, there is great potential to improve access to mental health care, reduce stigma, and bridge gaps in service delivery, ultimately contributing to a more inclusive and efficient psychiatric care system. Given that this study focused on usability and patient satisfaction with telephone-based psychiatric consultations in a single tertiary care setting, future research could explore comparative studies between telephone-based and in-person consultations to assess differences in clinical outcomes and therapeutic alliance.

## Data Availability

The original contributions presented in the study are included in the article/Supplementary Material, further inquiries can be directed to the corresponding author.
