# Correction: Assessing usability and satisfaction of telephone-based psychiatry consultations in Oman: a cross-sectional study

**DOI:** 10.3389/fdgth.2025.1657599

**Published:** 2025-07-11

**Authors:** Tamadhir Al-Mahrouqi, Fatema Al-Sabahi, Mohammed Al-Alawi, Munira Al Rubkhi, Marwa Al Abdali, Muna Al Salmi, Tharaya Al-Hashemi, Rahma Al Nuumani, Fatma Al Balushi, Rashid Al Zaidi, Samir Al-Adawi, Sachin Jose, Hassan Mirza

**Affiliations:** ^1^ Department of Behavioral Medicine, Sultan Qaboos University Hospital, College of Medicine and Health Sciences, Sultan Qaboos University, Muscat, Oman; ^2^ College of Medicine and Health Sciences, Sultan Qaboos University, Muscat, Oman; ^3^ Department of Psychiatry, Al Masarra Hospital, Ministry of Health, Muscat, Oman; ^4^ Research Department, Oman Medical Specialty Board, Muscat, Oman

**Keywords:** patient satisfaction, usability, health care accessibility, telehealth, telepsychiatry, psychiatric consultation

Correction on Assessing usability and satisfaction of telephone-based psychiatry consultations in Oman: a cross-sectional study By Al-Mahrouqi T, Al-Sabahi F, Al-Alawi M, Al Rubkhi M, Al Abdali M, Al Salmi M, Al-Hashemi T, Al Nuumani R, Al Balushi F, Al Zaidi R, Al-Adawi S, Jose S and Mirza H (2025). Front. Digit. Health. 7:1563180. doi: 10.3389/fdgth.2025.1563180

Affiliation “Department of Behavioral Medicine, Sultan Qaboos University Hospital, College of Medicine and Health Sciences, Sultan Qaboos University, Muscat, Oman” was erroneously given as “Department of Behavioral Medicine, Sultan Qaboos University Hospital, Muscat, Oman”.

The original version of this article has been updated.

